# Supplementary material for: Improvement in the Identification Technology for Asian Spongy Moth, Lymantria dispar Linnaeus, 1758 (Lepidoptera: Erebidae) Based on SS-COI
Source: Insects. 2023 Jan 16;14(1):94. doi: 10.3390/insects14010094 (PMC9867181; doi:10.3390/insects14010094)
Supplement: Supplementary file 1 [file insects-14-00094-s001.zip › insects-2064803-supplementary.pdf]

Supplementary Material

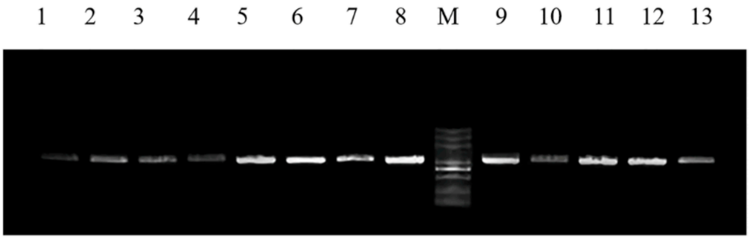

Figure S1 Agarose gel electrophoresis of PCR products amplified from DNA extracted from four species. M: DNA marker; Lanes1–2: Lymantria apicebrunnea; Lanes3–5: Lymantria monacha; Lanes 6–8: Lymantria xylina; and Lanes 9–13: Asian spongy moth.

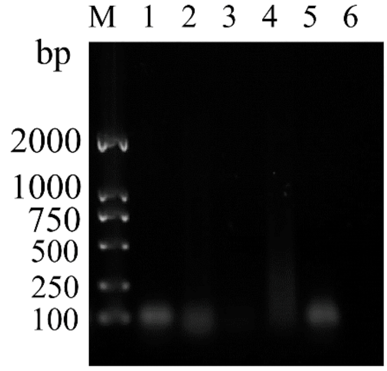

Figure S2 Agarose gel electrophoresis of the unsuitable annealing temperature for ASMF/ASMR. M: DL2000 DNA marker; Lanes 1–6 indicate the following annealing temperatures: 48°C, 47°C, 57°C, 58°C, 59°C, and 60°C, respectively.
